# THE VHA’S GERIATRIC SCHOLARS PROGRAM IMPACT ON DEVELOPING AND DISSEMINATING CLINICAL INNOVATIONS

**DOI:** 10.1093/geroni/igae098.0841

**Published:** 2024-12-31

**Authors:** B Josea Kramer, Judith Howe, Marianne Shaughnessy

**Affiliations:** Chair; Co-Chair; Discussant

## Abstract

The Geriatric Scholars Program is an enterprise-wide VA workforce development project that has resulted in significant clinical improvements and innovations in care of older Veterans. The program has invested in the interdisciplinary primary care workforce with discipline-specific core training in geriatrics and in quality improvement. Each of the more than 1500 Scholars to date implements a quality improvement project to demonstrate transfer of new knowledge and skills in the care of older Veterans. Ove 15,000 VHA clinical employees regularly participate in interdisciplinary webinars, team training workshops, practica, and self-directed learning opportunities. There are over 5000 users of the resource website, www.gerischolars.org. This symposium explores some of the direct impacts of continuing professional education on the clinical care of older Veterans. Several program components will be discussed, including replication in Indian Health Service clinics, outcomes for caregivers of older Veterans, practice change for geropsychologists, and how the program’s quality improvement mentored program for Geriatric Scholars enhances outcomes for patients in VHA. The Geriatric Scholars Program has 15 Geriatric Research, Education and Clinical Center (GRECC) partners in its network that have worked together for over 15 years to build capacity in the VHA healthcare workforce through innovative education and clinical approaches. The national GRECC Director in VHA will serve as discussant for what promises to be a lively exchange of ideas and opportunities for replication.

